# The microfoundations of physicians’ managerial attitude

**DOI:** 10.1186/s12913-021-06210-z

**Published:** 2021-03-04

**Authors:** Fausto Di Vincenzo, Daria Angelozzi, Federica Morandi

**Affiliations:** 1 Department of Economic Studies, G. d’Annunzio University of Chieti-Pescara, Viale Pindaro 42, 65127 Pescara, Italy; 2 Abruzzo Regional Health Agency, Via A. Monti 9, 65127 Pescara, Italy; 3 Department of Management, Catholic University of Sacred Heart, Largo F. Vito 1, 00168 Rome, Italy

**Keywords:** Middle managers, Managerial attitude, Microfoundations, Doctor-managers, Narcissism, Organizational identification, Specialization

## Abstract

**Background:**

Accountable care has profoundly changed the organizational models adopted by health care organizations and, consequently, the skill set required for doctor-managers who have become middle managers and must deal with the operational management of their units. The aim of this study was to identify the psychological microfoundations (i.e., traits) of physicians’ managerial attitude. Specifically, we analysed the roles played by narcissism, specialization choices and identification with the organization.

**Methods:**

We collected primary data on a population of ward unit heads in the Italian National Health Service. A logistic regression model predicting the levels of managerial attitude was employed.

**Results:**

The results indicate that high levels of narcissism and identification with the organization are related to higher managerial attitude (instead of clinical attitude). Additionally, we found that physicians with a technique-oriented specialization present a higher probability of manifesting managerial attitude (in comparison to clinical attitude).

**Conclusions:**

Hospital managers can benefit from the use of these findings by developing a strategic approach to human resource management that allows them to identify, train and select the right mix of technical knowledge and managerial skills for middle-management roles.

**Supplementary Information:**

The online version contains supplementary material available at 10.1186/s12913-021-06210-z.

## Background

Accountable care has changed the organizational arrangements adopted by health care organizations and, consequently, the skill set required for doctor-managers who have become middle managers and must deal with the operational management of their units [1]. An appropriate example in the literature is represented by unit heads, who are physicians tasked with a managerial role [2]. Unlike clinical directors, who are responsible for the general vision of an entire organization, unit heads act as a link between the operational and strategic levels of a hospital [3]. In their hybrid roles, they are responsible for clinical and managerial activities, including technology management, the control of clinical mistakes, the management of discharge programmes, budget negotiations, and the evaluation of results [4].

By taking into account the behavioural perspective, doctors in middle-management positions have been reported to face substantial difficulties attempting to perform the tasks assigned [5]. Specifically, doctor-managers are characterized as lacking managerial skills, which refers to the limited use of acquired competencies, and as lacking interest in managerial issues, which is related to scarce knowledge about the roles and tasks within organizational structures [6]. Moreover, they experience time pressures on their ongoing clinical activities [7], which are unpredictable and heterogeneous due to the managerial duties assigned.

This evidence raised questions about which professional model makes these new hybrid middle-management roles more effective [1, 7]. Empirical evidence suggests that despite professional competencies, technical knowledge and expertise play pivotal roles [8, 9]. Effectiveness also strongly depends on individual traits and managerial attitude, which act as important drivers in the transition of professionals into managerial positions [10, 11]. In this paper, we employ the construct of managerial attitude as a useful concept to explain why some doctor-managers are more effective at anticipating, interpreting and responding to challenging environments than others.

Research addressing physicians’ managerial attitude is emergent and growing. Certain personality traits have been associated with better individual performance, career success and professional attainment [12–17]. However, most of these studies focused on the consequences of managerial attitude, and we found a dearth of studies analysing its antecedents (i.e., how physician managerial attitude originates and develops). In other words, there is a shortage of studies that explore the so-called microfoundations of managerialism in healthcare [18, 19]. The relevance of a study on the microfoundations of physicians’ behaviour is also related to the knowledge gap generated by previous studies, which have focused mostly on the traits and behavioural skills of top figures such as chief executive officers or clinical directors [20]. Moreover, there is very little empirical evidence related to mid-level management roles, which are becoming crucial in the performance and accountability of health care organizations. The aim of this article is to address these research gaps by formulating specific research hypotheses and testing them using the middle managerial roles that doctor-managers perform within hospitals.

## Hypotheses development

A first individual trait that has received scarce attention in the health care management literature is narcissism. Narcissism is broadly defined as an exaggerated yet fragile self-concept of one’s importance and influence [21]. Narcissists are naturally attracted to organizational leadership roles due to their desire to leave behind a grand, admirable legacy of achievement [22], especially when the context is particularly challenging [23]. Ignoring the negative characteristics associated with narcissism and focusing on the positive narcissistic characteristics, scholars have demonstrated that healthcare professionals are equipped with the right amount of psychological capital to be able to moderate and positively use the effects of managers’ narcissism [24].

Generally, narcissism is strongly related with the achievement of high organizational successes [25]. There are several examples in the literature about the connection between narcissism and challenging managerial tasks, including the relationship between narcissism and the degree of corporate social responsibility of an organization [26], internationalization decisions [27], and earning behaviours [28]. When managers are equipped with a certain amount of narcissism, they can achieve an effective managerial attitude. This is why we considered narcissism as the first of the microfoundations of managerial attitude in our analysis and hypothesize the following:

- HP1: *The higher the narcissism trait of doctor-managers is, the higher their managerial attitude*.

Another individual psychological trait related to the self-concept and that has received more attention recently – but nevertheless requires further study – is physicians’ organizational identification with their hospital [29]. Organizational identification concerns the extent to which an individual’s self-concept contains attributes identical to those of the perceived organizational identity. The strength of an individual’s organizational identification depends on how well the image of an organization preserves the continuity of the individual’s self-concept, provides distinctiveness, and enhances self-esteem [30]. When individuals adopt the values and goals of the organization, they develop decision-making approaches complementing the values and goals held by the organization [31]. From a managerial perspective, identification is advantageous since it ensures that employees’ decisions will be made in the best interest of the organization, even in the absence of supervision [32].

From a cognitive perspective, the shared interests between employees and their organization allow individuals to feel and recognize themselves as a part of it [33]. In addition, identification with one’s organization introduces a feeling of pride. For example, when individuals highly identify with their organization, they will positively contribute to its success and perform extra role behaviours [34]. This is expected from doctors serving in managerial positions that are asked to be good doctors and good managers at the same time [16, 35]. For this reason, we considered organizational identification as the second microfoundation of managerial capabilities and hypothesize the following:

- HP 2: *Highly identified doctor-managers recognize the importance of the managerial aspect of their work, thus showing a higher degree of managerial attitude*.

Finally, the third microfoundation we considered in our analysis is the specialization of physicians tasked with managerial roles. Within modern healthcare systems, due to the entrepreneurial profile of hospitals, competition for resources and patients is high [36]. For these reasons, physicians serving in hybrid roles are increasingly required to reach high levels of performance. To achieve better results, physicians need to develop and to fulfil their value through their activities. Therefore, if their work is more oriented to the intrinsic values (creativity and training) than to the extrinsic ones (earnings and career), their focus will be more oriented towards the clinical rather than the managerial side and vice-versa. Diversified value structures based on an individual’s background is a topic of particular interest in the field of organizational behaviour, as observed by Shapira and Griffith [37] who discussed the misalignment of work values in relation to individuals’ professional specialties. Several prior studies focused on the relationship between medical specialty choices and working behaviours [38]. They explored the correlation between a medical specialty and career decisions, interest in the status and prestige of a work position [39], the establishment of good relationships with colleagues and “self-direction” in their own work [40].

Medical specialties can be classified into two broad categories: person-oriented and technique-oriented [41], as reported in Table 1.

**Table 1:** Distribution of the specialties between “Groups P and T”

	Group P	Group T
Pathology		x
Cardiovascular diseases	x	
General surgery		x
Diagnostic and surgical endoscopy		x
Surgical emergency		x
Maxillofacial surgery		x
Thoracic surgery		x
Vascular surgery		x
Haematology	x	
Endocrinology	x	
Diabetes and metabolism specialization	x	
Geriatric medicine	x	
Infectious diseases	x	
General medicine	x	
Nephrology and dialysis		x
Neurology		x
Eye care specialist		x
Dentistry		x
Orthopaedic and traumatology		x
Obstetrics and gynaecology	x	
Ear and throat specialist		x
Paediatrician	x	
Urology		x
Anaesthesia, Reanimation, and Intensive Care		x
Rehabilitation and physical medicine	x	
Gastroenterology	x	
Oncology	x	
Pulmonology		x
Radiology		x
Radiology		x
Radiotherapy		x
Rheumatology	x	

In terms of their approach to work, person-oriented specialties focus on the holistic view of the patient and tend to emphasize the relational and empathic approach with the patient. In contrast, technique-oriented specializations are more focused on the technical skills, tools, and technologies that are needed to provide healthcare processes and activities. In terms of their appreciation of the work, technique-oriented physicians are more interested in status, prestige and career, thus appearing to be more in line with a managerial profile in comparison to person-oriented physicians. For these reasons, we hypothesized the following:

- HP 3: *Technique-oriented specialized physicians have a higher managerial attitude than their person-oriented specialized colleagues*.

## Methods

### Research setting and data collection

Empirical analyses were carried out in hospital organizations within the healthcare service of Abruzzo in central Italy. The healthcare system of Abruzzo is part of the Italian National Health Service, a publicly funded universal health system that provides single-payer universal coverage. The central Italian government defines the core benefit packages and oversees the basic coverage being provided to the entire population, and each region is responsible for administering and organizing community healthcare services for its population.

The analysis draws on a range of rich data. The data were collected from questionnaires that were provided to public hospitals operating within the target region only. By reading the strategic plan and organizational chart of each public hospital, we were able to identify the number of ward units, which constituted our study sample size. Each unit has a director who is formally appointed as head of the unit, and is responsible for the organization of health services and has both clinical and managerial responsibilities, including negotiating the goals of the unit with the general manager of the hospital.

The data collection was made possible through the collaboration of a wide range of actors, including CEOs, medical directors, and regional representatives of unit head unions. The questionnaire was developed in Italian specifically for this study. The English language version of the questionnaire is provided as Additional File 1.

The questionnaire administered to the heads of ward units encompassed different sections. The first section collected personal data, including the respondent’s age, hospital affiliation, seniority, sex, type of medical specialization achieved, and his or her training programmes and career paths prior to their current assignment. The second section was designed to capture the perceived level of organizational identification. The third section was a self-reported measure of narcissism. Finally, the fourth section examined managerial attitude.

Of the 332 unit heads operating within the 17 public hospitals in the region, 126 agreed to be interviewed (overall response rate of 37.95%). All interviews were conducted in person and on site, lasting from 17 min to 1 h, middle managers were asked to respond with the first answer that comes to mind, and they were not allowed to use or consult reports and smartphones. During the interviews, participants received a description of the survey’s purposes and they were asked to sign an informed consent, in accordance with applicable Italian data protection laws. If participant did not sign the consent, the interview was automatically stopped.

### Variables

#### Dependent variable

The dependent variable, namely, managerial attitude, is a dummy variable. Developed according to Cicchetti’s (2005) work, it is based on 16 items concerning the bundle of managerial traits desirable for unit heads. It includes a number of dimensions such as autonomy (“My work is independent of that of others”), action-orientation (“I’m action oriented and I do not need to schedule my activities in advance”), and orientation to social interactions (“I’m prone to maintain a number of social and work interactions”). A 6-point Likert scale, wherein more positive values represented higher managerial traits while lower values represented a higher clinical orientation, was used to answer the questions. In the attempt to have a synthetic index, we calculated a mean value for each respondent. Successively, we computed the median value of the sample (4.38), which was used as a cut-off. When the individual mean value was less than 4.38, we assigned a value = 0, thus indicating a clinical-oriented attitude; meanwhile, we assigned a value = 1 when the individual mean value was over 4.38, thus indicating the presence of managerial attitude.

#### Explanatory variables

##### Organizational identification

Employees’ identification with their organization was measured using Bergami and Bagozzi’s [33] approach. This approach uses a graphical instrument with two circles that vary in their overlap, ranging from being apart from each other to overlapping completely (with the latter indicating strong identification) and consists of 7 possible answers.

##### Narcissism

We measured physicians’ narcissism trait through a single item question derived from the work of Konrath et al. [42]. Although previous studies have employed proxies of narcissism based on publicly available information [23] or self-reported multi-item scales [25], in this study, we decided to follow previous scholars’ approach (among other Konrath [42] e Ames et al. [43]) who suggested this measurement within knowledge-intensive organizations where professionals are employed. Specifically, authors affirm that when respondents are professionals (such as physicians) with limited time available, and when the questionnaire must necessarily have a limited number of questions, a single item dimension for inspecting narcissism can be adopted. Following this suggestion, we asked to our sample to express their degree of accordance with the statement “I am more capable than other people”. We assessed their level of agreement with this statement employing a Likert scale ranging from 1 = completely disagree to 5 = completely agree.

##### Technique-oriented specialization

This is a dummy variable. Based on the classification provided by the literature about person-oriented specializations and technique-oriented specializations [38, 41], we established two groups of respondents: “Group P” (person oriented) and “Group T” (technique oriented). Table 1 provides the distribution of the specialties between the two groups. We assigned a value of 1 to physicians with a technique-oriented specialization and 0 to the physicians with a person-oriented specialization. Person-oriented physicians were used as controls just within Model 3.

##### Sex

We assigned value 1 for male respondents and 0 for female respondents.

##### Academic experience

This is a dummy variable used in order to assess respondents’ previous experience as scholars within medicine and surgery courses. A value of 1 was assigned if respondents reported having these experiences and 0 otherwise. During academic experiences, scholars may enlarge their patterns of knowledge about healthcare systems. Currently, medicine and surgery academic courses are multidisciplinary since their focus is not only on a clinical vision of healthcare organizations and systems but also on managerial implications [44].

##### Tenure

A continuous measure of the length of time (in years) that a unit head has been in charge was employed as a control variable.

Table 2 presents the descriptive statistics for the independent variables and the pairwise correlation indices.

**Table 2:** Descriptive statistics of the independent variables and their pairwise correlations

		Mean	Std dev	1	2	3	4	5	6	7	8
1	Managerial attitude	.531	.500	–							
2	Sex (1 = male)	.873	.334	−.023	–						
3	Academic experience	.325	.470	.040	.112	–					
4	Tenure	8.38	6.85	−.071	.120	.172	–				
5	Narcissism	.619	.487	.213*	−.004	−.013	.144	–			
6	Organizational identification	5.71	1.37	.117	−.062	−.151	.112	−.068	–		
7	Technique-oriented	.595	.429	.165	.122	−.048	.-036	.085	−.148	–	
8	Person-oriented	.404	.492	−.165	−.122	.048	.036	−.085	.148	−1	–

### Estimation technique

Due to the dichotomous nature of the dependent variable, managerial attitude, we performed a logistic regression [45]. We analysed three models: the first model named “Model 1” includes only the control variables. Model 2 adds the variables narcissism, organizational identity and technique-oriented specialization to Model 1. Finally, to demonstrate how managerial attitude decreases according to the different specialization typologies, we introduced an alternate technique-oriented specialization variable, the person-oriented specialization variable, in Model 3.

## Results

The empirical results of the logistic regression estimations are presented in Table 3. Model 1 reflects the inclusion of only the control variables. Model 2 adds all the explanatory variables to Model 1. As expected, narcissism is positively related to the dependent variable (β = 1.06, *p* ≤ .01), indicating that the higher the narcissism trait of physicians is, the higher their probability to present a managerial attitude instead of a clinical attitude. Organizational identity increases respondents’ probability of presenting a managerial attitude instead of a clinical attitude, (β = .325, *p* ≤ .05). This indicates that physicians who identify with their organizations’ values and goals are more likely to express a managerial attitude, thus showing awareness of the challenges associated with the managerial role that the organization entrust to them and which they perform at the expense of the more traditional approach in which physicians mainly focus on the clinical side of their work. Finally, within Model 2, the technique-oriented specialization is positively related to the dependent variable (β = .807; *p* ≤ .05). This result indicates that physicians with a technique-oriented specialization are more likely to express a managerial attitude than a clinical attitude. As expected, since a person-oriented specialization is the opposite of a technique-oriented specialization, physicians with a person-oriented specialization have a lower probability of expressing a managerial attitude compared to a clinical attitude (β = −.807; *p* ≤ .05).

**Table 3:** Logistic regression results

Dependent variable = Managerial attitude = 1			
	Model 1	Model 2	Model 3
Sex (1 = male)	−.124	−.195	−.195
	*(.54)*	*(.57)*	*(.57)*
Academic experience = 1	.244	.522	.522
	*(.39)*	*(.42)*	*(.42)*
Tenure	−.023	−.043	−.043
	*(.02)*	*(.02)*	*(.02)*
Narcissism	–	1.06**	1.06**
		*(.40)*	*(.40)*
Organiz. identification	–	.325*	.325*
		*(.156)*	*(.156)*
Technique-oriented = 1	–	.807*	–
		*(.39)*	
Person-oriented = 1	–	–	−.807*
			*(.39)*
# Observations	126	126	
Nagelkerke Likelihood	.011	.154	

*p* ≤ 0.001***, *p* ≤ 0.01**, and *p* ≤ 0.05*

## Discussion

Healthcare reforms in publicly funded universal health systems must revise hospital organizational roles. Important tasks have been delegated to middle managers who are thought to connect the strategic and the operational levels of an organization [2]. In hospitals, middle managerial roles are occupied by doctor-managers who must navigate dual roles (managerial and clinical) [46]. These professionals are experiencing dramatic changes in their working conditions [1, 7], and empirical evidence suggests that the effectiveness of such changes strongly depends on their abilities [8, 9]. Although clinical technical knowledge and expertise are still fundamental [1, 20], physicians engaged in managerial roles also need to possess other individual skills, namely, managerial capabilities [10, 11]. This is a useful concept to explain why some managers have more effective capabilities at anticipating, interpreting and responding to challenging environments than others.

The literature shows that there is a growing interest in studies analysing the psychological microfoundations of physicians’ managerial attitude. In fact, within healthcare contexts, improvements are driven by individuals and their abilities, which the literature refers to as microfoundations [47]. By following this perspective and considering the specificity of healthcare organizations, this study has focused on three individual microfoundations—narcissism, organizational identification, and specialization—as correlates of managerial attitude.

This research found that the managerial attitude of doctor-managers is positively affected by the presence of a narcissistic trait. This extends the results of previous management studies to the healthcare sector [42] and suggests that managers who are equipped with the right amount of narcissism can help their organization to solve complex and challenging situations. This result also highlights an aspect often not considered during the recruitment processes and the definition of the career paths of middle managers within hospital organizations.

This research also found that organizational identification is closely related to managerial attitude. The results show that the higher that one’s organizational identification is, the higher the likelihood that a given physician will develop a managerial attitude. This extends the results of Salvatore et al.’s [29] study on organizational identification. This research suggests that when doctor-managers actively take part in the strategic goals of the organization to which they belong and they have a strong focus on the strategic and entrepreneurial activities in line with their double role, this in turn implies the development of the managerial side of their work. Managerial attitudes can increase when doctor-managers understand, share and identify with the strategic goals of the organization.

Finally, this research found that a technique-oriented specialization for physicians positively affects the level of managerial attitude. By virtue of the principle of value congruence, we confirm that physicians need to meet their needs within the workplace, especially when they are asked to perform extra role behaviours [35]. There is a strong relationship between a medical specialization and the meaning attributed to the role of “doctor” [38, 40]. Person-oriented physicians are more interested in the clinical and social aspects of the work. Their colleagues that specialize in technique-oriented fields are instead more interested in the status aspects of the work, such as the acknowledgement of their colleagues. Technique-oriented physicians are more likely to accept managerial responsibilities because these responsibilities facilitate their desire to achieve a status in their organization that gratifies their professionalism and provides access to more sophisticated, strategic, and costly technologies [39].

The present study adds a fresh perspective to the debate of medical management and provides new insights into how to develop managerial attitude among hospitals’ middle managers. However, the study has some limitations that provide opportunities for future research. First, we considered physicians working in public healthcare organizations. The same study assessing the managerial attitude of employees within a private context could yield different results. Second, we surveyed physicians staffed within hospitals located in a single region in the Italian NHS. Although Italian regions may differ for some organizational features, there is a strong central government harmonization of human resource management practices, especially as concern selection and hiring procedures, careers paths and training programs aimed at improving managerial skills. Therefore, this makes the considerations exposed for this specific sample certainly generalizable to the other Italian regional contexts. Moreover, with due caution, our results can be extended also to other highly centralized National Health Systems such as the UK’s one, while further research is needed to validate our results within other contexts and health care systems.

Third, we measured physicians’ narcissism through a self-reported single item question. Although this approach is commonly used in the literature, future studies will have to confirm whether our results are also valid with the application of multi-item scales and/or combining self-reported data with publicly available information. Also, we do not consider the “dark side” of narcissism, and thus we have no information on the negative consequences connected to the presence of this behavioural trait. Fourth, we do not control for a performance index in order to understand if the presence of a managerial attitude within doctor-managers may improve their effectiveness and efficiency in achieving individual goals, as well as the results achieved by the organization. Finally, we use a cross-sectional approach, and we do not consider any changes over time. Further research is encouraged to explore the effects of microfoundations on physicians’ managerial attitude in order to extend the application to longitudinal models and other health care settings.

Despite these limitations, we believe that the problem addressed remains of general interest and relevance for hospital managers and health policy makers.

## Conclusions

This study provides novel insights into understanding the microfoundations of managerial attitude and highlights a nascent research avenue with diverse development opportunities. Physicians in middle-management positions must deal with the operational management of their units; to perform the assigned tasks well, they need to develop an adequate managerial attitude. Among the antecedents of managerial attitude, some psychological traits of the individual, such as narcissism, identification with the organization and the type of specialization, play fundamental roles.

This study has implications for hospital managers. In the recruiting phase, many organizations focus solely on clinical skills without considering candidates’ managerial skills and psychological traits. In addition, many hospital organizations do not carry out structured analyses of physicians’ training needs and/or do not have resources available to invest in training activities, leaving training to an individual’s own initiative without specified content or objectives. Finally, the choices related to middle-management positions are often tied more to seniority than to aptitude for carrying out that specific role. Our study supports the development of a strategic approach to human resource management that allows one to identify, train and select the optimal mix of knowledge and managerial skills to cover a middle-management role.

## Supplementary Information

**Additional file 1.**

## Data Availability

The datasets used and/or analysed during the current study are available from the corresponding author on reasonable request.
