# Supplementary material for: The microfoundations of physicians’ managerial attitude
Source: BMC Health Serv Res. 2021 Mar 4;21:199. doi: 10.1186/s12913-021-06210-z (PMC7934558; doi:10.1186/s12913-021-06210-z)
Supplement: Supplementary file 1 — Additional file 1. [file 12913_2021_6210_MOESM1_ESM.docx]

**Section 1 - Demographic data**

- Age
- Hospital affiliation
  - Name of the Department
  - Name of the Unit
- Seniority as Head of Unit
- Sex
- Medical Specialization
- Training programs on Health Care Management attended
- Previous roles covered before the actual position

**Section 2 - Organizational identification**

Please show the degree of your organizational identification. Within each row circles represents your individual identity (Me), and those of organization (Organization). Select the solution better representing your organizational identification.

|  | | Me | Organization | (Identity) | |  |
| --- | --- | --- | --- | --- | --- | --- |
|  |  | | | |  | |
| A |  | | | | Far | |
| B |  | | | | Near, but separated | |
| C |  | | | | Very light overlapping | |
| D |  | | | | Light overlapping | |
| E |  | | | | Moderate overlapping | |
| F |  | | | | High overlapping | |
| G |  | | | | Very high overlapping | |
| H |  | | | | Complete overlapping | |

**Section 3 – Narcissism**

Please indicate the degree of agreement with the following statement where (1: Completely disagree, 5: Completely agree)

- I am more capable than other people

**Section 4 - Managerial attitude**

**Please indicate the degree of agreement with the following statement where (1: Strongly disagree, 6: Strongly agree)**

- I am typically a planner: I plan my activities in advance
- I’m prone to maintain a number of social and work interactions
- I am led to "anticipate" events and in some way to induce them
- When performing a task, I don't feel the need to have instant gratification for the work done
- I prefer to share goals and work with others: collaboration is at the basis of success
- My work contributes to the achievement of overall goals
- My concern is to carry out my work in compliance with the procedures and rules set by the organization
- When I meet someone, I generally present myself as belonging to the institution I am currently working for
- I’m action oriented and I do not need to schedule my activities in advance
- I’m prone to the one-to one interaction
- I promptly react to the events
- When performing a task, I want quickly to see the result
- I prefer to work alone and to assume my own responsibilities
- My work is independent of that of others
- My concern is to take care about patients
- When I meet someone, I generally present myself as a doctor
